# Forum: Climate, Ecological, and Social Costs of Livestock Grazing on Western Public Lands

**DOI:** 10.1007/s00267-023-01853-6

**Published:** 2023-07-14

**Authors:** J. Boone Kauffman, Robert L. Beschta, Peter M. Lacy, Marc Liverman

**Affiliations:** 1 Illahee Sciences International, Corvallis, OR 97330 USA; 2 Department of Fisheries, Wildlife and Conservation Sciences, Oregon State University, Corvallis, OR 97331 USA; 3 Department of Forest Ecosystems and Society, Oregon State University, Corvallis, OR 97331 USA; 4 Oregon Natural Desert Association, Portland, OR 97211 USA

**Keywords:** Climate mitigation, Climate crisis, Greenhouse gas emissions, Livestock grazing, Public lands, Rangelands

## Abstract

Grazing by domestic livestock is the most widespread use of public lands in the American West (USA) and their effects on climate change and ways to mitigate those effects are of interest to land managers, policy makers, and the broader public. Kauffman et al. (2022a) provided a meta-analysis of the ecosystem impacts, greenhouse gas (GHG) emissions, and social costs of carbon (SCC) associated with livestock grazing on public lands in the western USA. They determined that GHG emissions from cattle on public lands equaled 12.4 million t CO_2_e/year. At the scale of land use planning utilized by federal agencies, GHG emissions associated with allocated livestock numbers will typically exceed US Environmental Protection Agencies’ reporting limits (25,000 t) for certain industrial greenhouse gas emitters. As such, these are essentially unreported sources of GHG emissions from public lands. Using the US government’s most recent SCC estimate of $51/t, Kauffman et al. (2022a) determined the total SCC of cattle grazing on public lands to be approximately $264–630 million/year. However, recent advances in the determination of SCC reveal this is to be an underestimate. Using the latest science results in an estimated SCC of $1.1–2.4 billion/year for grazing on public lands. Furthermore, the SCC borne by the public exceeds the economic benefits to private livestock permittees by over $926 million/year. Cessation of public lands grazing is an environmentally and economically sound mitigation and adaptation approach to addressing the climate crisis; an approach that will also facilitate restoration of the myriad of ecosystem services provided by intact wildland ecosystems.

## Introduction

Livestock grazing is the most extensive land use on federally managed public lands in the American West (Beschta et al. (2012)); hence, attention to ways in which livestock grazing contributes to climate change is warranted. These livestock, mostly cattle, influence climate change in three profound ways: (1) they are significant sources of greenhouse gases (GHG) through enteric fermentation and manure deposition (Gerber et al. 2013; USEPA 2018); (2) they defoliate native plants, trample vegetation and soils, and exacerbate the spread of exotic plant species, resulting in a shift in landscape function from important carbon sinks to sources of GHG (Kauffman et al. 2022a); and (3) their grazing intensifies the effect of climate change on ecosystems by creating even warmer and drier conditions (Kauffman et al. 2022a, b).

To address the climate crisis, federal agencies in the USA are tasked with reducing GHG emissions. These agencies have recognized that it is essential to account for the benefits of reducing climate pollution, by capturing the full costs of GHG emissions as accurately as possible and by taking global damages into account [(e.g.,) Executive Order 13990 (2021) and Interior Secretarial Order 3399 (2021)]. Included are agencies that manage the vast public lands and resources of the American West - mainly the Bureau of Land Management (BLM) and the US Forest Service (USFS). The standard cost-benefit measure and a key metric for assessing climate policy is the social cost of carbon (SCC), which estimates in dollars the long-term damage done by GHG in a given year (Rennert et al. 2022; Aldy et al. 2021; USDI 2021). SCC calculations draw on climate science, economics, demography, and other disciplines and are used by governments and other decision-makers in cost-benefit analyses.

Kauffman et al. (2022a) determined GHG emissions attributed to enteric fermentation and manure deposition originating from federally authorized domestic cattle grazing on public lands in the western US. The US Environmental Protection Agency (USEPA 2018) default values for beef cattle in the western US were used to calculate the enteric emissions from cattle using public lands. This was 100 kg methane (CH_4)_/year for cows and 11 kg CH_4_/year for calves. Methane emissions from manure (2.4 kg CH_4_/year/animal) were from Wolf et al. (2017) and nitrous oxide (1.4 kg N_2_O/year/animal) were from IPCC (2006).

The relative capacity of a GHG to trap heat in the global climate system over a given time frame compared to that of carbon dioxide is expressed as its global warming potential (GWP). There is somewhat of a disconnect between the GWP and the economic valuation of the SCC of CH_4._ The current SCC of CH_4_ is $1500/t and $51/t for CO_2_ [Interagency Working Group on Social Cost of Greenhouse Gases IWG (2021)]. The SCC for CH_4_ is about 29 times that of the valuation of the CO_2_ equivalence (CO_2_e). However, the GWP of CH_4_ is 34 for a 100-year GWP and 86 for the 20-year GWP (IPCC 2013). Thus, the use of SCC of CH_4_ rather than conversion to CO_2_ equivalence results in a slightly lower SCC estimate using a 100-year GWP and a dramatically lower SCC when using a 20-year GWP.

In terms of enteric fermentation and manure deposition contributions to climate change, Kauffman et al. (2022a) determined that one cow-calf pair grazing for one month (an “animal unit month” or AUM) on public lands, produces 391 kg CO_2_e using a 100-year global GWP and 875 kg CO_2_e using a 20-year GWP. A mean of 15.4 million AUMs of total livestock use occurred annually from 2009–2016 on federally managed public lands, of which cattle account for over 91% of all domestic animals grazing on BLM and USFS lands in the western USA. Thus, for the most recent 10-year period in which data are available, an average of 8.0 million and 6.1 million AUMs of cattle grazed on public lands managed by the BLM and the USFS, respectively (USDA 2021, USDI 2021, Kauffman et al. 2022a).

Using the US government’s 2021 SCC values, Kauffman et al. (2022a) calculated the SCC of an AUM was $19-$45/AUM (Table 1). Based on these values, Kauffman et al. (2022a) determined that the SCC of livestock grazing on public lands in the western USA ranged from $264 to 630 million/year. However, the calculated SCC of Kauffman et al. (2022a) was most likely a major underestimation of the true social costs of livestock on public lands as their estimate only included emissions from enteric fermentation and manure deposition. It did not include the increased carbon emissions due to desertification, degradation, and land cover change that are associated with livestock activities. For example, Kauffman et al. (2022a) reported the cessation of livestock grazing in riparian areas alone could result in the sequestration of 2.2 million t C/year (8.1 million t CO_2_e/year). This is equivalent to a SCC of $413 million/year. They also reported an estimated 24.7 to 80.2 million t CO_2_e per year have been lost through purposeful conversion of native ecosystems to those dominated by exotic species representing an additional annual SCC of $1.3–4.0 billion.

**Table 1:** The social cost of carbon (SCC in $USD) per animal unit month (AUM) arising from the enteric fermentation and manure deposition of cattle on public lands

Emission type and source	Rennert et al. (2022)		Interagency Working Group on Social Cost of Greenhouse Gases, United States Government (2021)		
	GWP 20	GWP100	N_2_O and CH_4_	GWP 20	GWP100
CH_4_ emission—fermentation	$147.17	$58.18	$13.88	$40.57	$16.04
CH_4_ emission—manure deposition	$3.18	$1.26	$0.60	$0.88	$0.35
Subtotal: social cost CH_4_ emission/AUM	$150.35	$59.44	$14.48	$41.45	$16.39
N_2_O emission—manure	$11.57	$12.86	$4.20	$3.19	$3.55
Total social cost/AUM	$161.92	$72.30	$18.68	$44.64	$19.93
Total social cost from cattle grazing public lands	$2.29 billion	$1.02 billion	$263.7 million	$630 million	$281 million

Results are based upon conversion of methane (CH_4_) and nitrous oxide (N_2_O) to carbon dioxide equivalents (CO_2_e) using 20 and 100-year global warming potentials (GWPs). We also report the SCC/AUM based upon published social costs of CH_4_ and N_2_O. Rennert et al. (2022) determined that the social cost of carbon is $185/t CO_2_e at a near-term risk-free discount rate of 2%. The Interagency Working Group on Social Cost of Greenhouse Gases (2021) determined costs based upon a 3% discount rate, resulting in $1500/t CH4, $18,000/t N_2_O, and $51/t CO_2_e. Total social cost of grazing from public lands is the sum from the average number of AUMs (cattle) grazing western public lands each year (Supplementary Information Tables S2 and S3)

Significant new information has recently emerged (Rennert et al. 2022; USEPA 2022) that suggests the SCC reported by Kauffman et al. (2022a) are considerable underestimates. These recent studies incorporated research regarding damages from four categories of climate change impacts: (1) premature deaths caused by extreme heat, (2) impacts on agricultural yields, (3) energy use in response to temperature changes, and (4) sea-level rise. They did not account for other climate change impacts with economic effects relevant to western public lands, such as increased incidences of wildfire and droughts. While decreased agricultural productivity was included in the estimate of SCC, declines in the capacity to sequester carbon (a negative feedback) were not.

Applying recent, peer-reviewed advances in climate, economic, and demographic science, (Rennert et al. 2022) calculated a new preferred mean SCC of $185/ t CO_2_ ($44–$413/ t CO_2_: 5%–95% range, 2020 US dollars) at a near-term risk-free discount rate of 2%. This preferred value is 3.6 times higher than the US government’s current value (the value utilized by Kauffman et al. 2022a). Thus, the calculated SCC reported by Kauffman et al. (2022a) represents a highly conservative, and now outdated estimate of the long-term climate damage resulting from livestock grazing. Even more recently, the US Environmental Protection Agency (US Environmental Protection Agency (2022)), proposed increasing the SCC to $190/t of CO_2_, using a 2% discount rate (Supplementary Information Table S1). Using the Rennert et al. (2022) figures, the SCC for federally authorized cattle grazing on public lands in the western USA is estimated to be $2.3 billion/year, based on a 20-year GWP and $1.1 billion /year based on a 100-yr GWP. By comparison, the grazing fees collected by federal agencies for public-land livestock grazing is about 1% of the SCC (Vincent 2019).

## Costs and Benefits of Grazing Livestock on Public Lands

The fact that federally authorized livestock grazing costs the US government far more than it collects through grazing fees is well documented (Vincent 2019). But, how do the economic benefits of livestock production compare with the SCC? Taylor et al. (2022a, 2022b, 2022c) estimated the economic effects of removal of grazing on federal lands for the states of Oregon, Idaho, and Wyoming, USA by calculating the direct economic impacts as well as the secondary economic impacts associated with livestock removal. Based upon their data, the direct economic decline due to livestock removal from public lands was equivalent to $27.50/AUM in Oregon, $35.11/AUM in Idaho, and $35.21/AUM in Wyoming. The social cost of carbon associated with GHG emissions only from enteric fermentation and manure deposition (i.e., $72–$166/AUM) greatly exceed the economic value of allowing cattle to graze on public lands (Supplementary Information Table S1, Table 1).

Taylor et al. (2022a, 2022b, 2022c) did not include the SCC of cattle grazing on public lands in their estimates of livestock values. Greenhouse gas emissions from enteric fermentation and manure deposition on public lands were 1.3 million t CO_2_e for Oregon, 1.6 million t CO_2_e for Idaho, and 2.1million t CO_2_e for Wyoming. Comparing the direct impacts of livestock removal with the SCC is most relevant since both only consider the influences of cattle on public lands. More complicated assessments comparing the direct and secondary costs of livestock removal with the potential benefits of their removal would require life cycle analyses that include responses and values of ecosystem services, such as improved water quality, water quantity, carbon sequestration, lowered erosion, pollination services, increased biological diversity, and esthetics (e.g., Fleischner 1994, Ohmart, Anderson (1986), Donahue 1999, Beschta et al. (2012), Kauffman et al. 2022a).

**Fig. 1:**
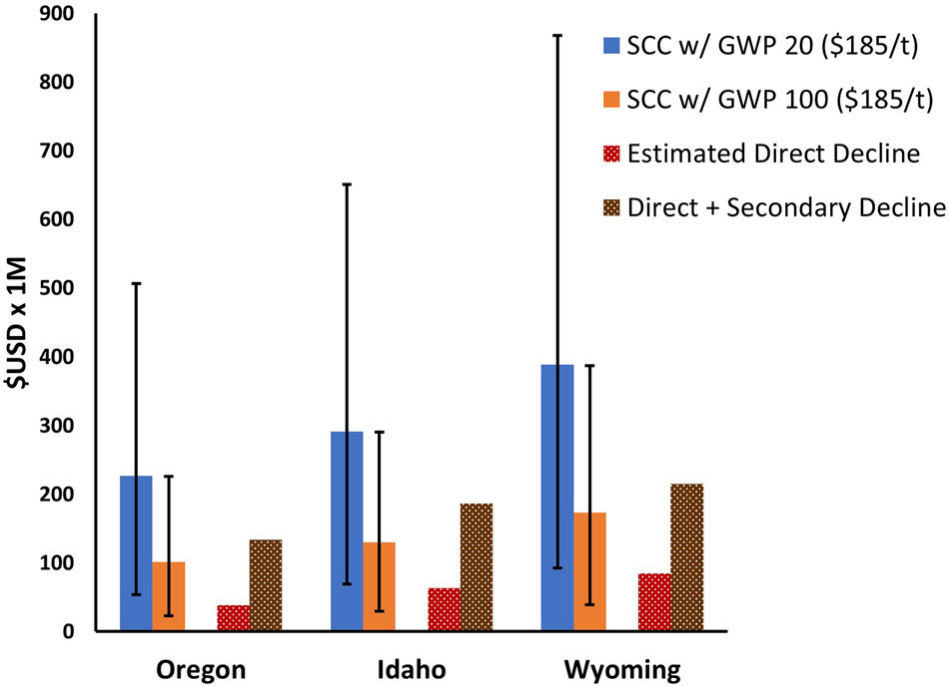
The social carbon cost (SCC, colored bars) arising from cattle grazing on federally managed public lands in three western states, along with the direct and secondary economic declines (thatched bars) that would arise from their removal. SCC values are based on costs of $185/t CO2e from Rennert et al. (2022) with associated uncertainty ranges (thin, capped vertical bars; $44–$413/ t CO_2_: 95% range, 2020 US dollars). The estimated economic declines of livestock removal are from Taylor et al. (2022a, 2022b, 2022c); uncertainties for these costs were not provided. Each of the different approaches to determining social carbon costs are consistently higher than direct economic losses from livestock removal suggesting a net societal economic benefit to removal. The SCC of livestock grazing, derived from a 20-year global warming potential (GWP) even exceeds the direct and secondary impacts of livestock removal

Using data provided by Taylor et al. (2022a, 2022b, 2022c) we determined the differences in the economic loss of cattle removal for public lands to that of the SCC of grazing cattle on public lands for a western state with significant public lands. For example, the 1.4 million AUMs of grazing on Oregon’s federally managed public lands results in GHG emissions of 1.2 million t CO_2_e. This represents an SCC of $63 million and $28 million/year (using the current federal government’s SCC with a GWP of 20 and 100 years, respectively Fig. 1). However, using updated SCC figures of Rennert et al. (2022) results in SCC estimates of $101million (100-year GWP) to $226 million (20-year GWP) per year for Oregon. By contrast, the economic impact of cattle removal (Taylor et al. 2022b) is $38.5 million/year. The social costs of carbon borne by the public for livestock grazing on federally managed public lands therefore far exceeds the economic benefits to the private livestock owners who utilize those lands.

Our analyses only pertain to cattle grazing on federally managed public lands and does not include the effects of cattle grazing on state lands or private lands. In Oregon for example, there are about 11,500 farms with beef cattle and about 2030 federal grazing permits. This suggests that about 83% of the livestock producers in Oregon only utilize private lands to raise their cattle. In 2017, the average grazing cost on state land in the West was $23.90/AUM and on private land was $23.40/AUM (Vincent 2019). On federally managed public lands, grazing fees are $1.35/AUM, thus representing a considerable subsidy to those livestock producers.

There are concerns that if cattle are eliminated from public lands, there will be no net loss of greenhouse gas emissions because they will simply shift impacts to private lands (the concept of leakage). Kauffman et al. (2022a) described that leakage to private lands would likely be minimal because of: (1) the enhanced carbon sequestration from recovering public lands following the removal of livestock; (2) the higher forage quality (digestible energy) and availability of methane reducing feed additives on private lands; and (3) dietary shifts away from beef.

The 12.4 million t CO_2_e/year of GHG emissions arising from federally authorized livestock grazing reported here is only for emissions from enteric fermentation and manure deposition and does not include the substantial carbon removals that would arise from ecosystem recovery following removal of livestock (Kauffman et al. 2022a). Nevertheless, these emissions far exceed reporting limits set by the USEPA (2023) for industrial facilities that similarly emit climate pollution into the atmosphere. The USEPA regulations require certain industrial greenhouse gas emitters to report to the agency if their action results in 25,000 t or more of CO_2e_ per year. Although this reporting program is not used directly as an emission control program, it nevertheless helps the USEPA and the public to understand where GHG emissions are coming from, and will improve the ability to make informed policy, business, and regulatory decisions (USEPA 2023). It requires about 2381 cattle (28,571 AUMs) on public lands to generate 25,000 t CO_2_e.

At the scale of land use planning utilized by federal agencies such as the different National Forests or BLM Districts, and where these agencies establish grazing numbers as required by law, GHG emissions from livestock often far exceed EPA reporting limits (Table 2). For example, in the Lakeview District, Oregon Resource Management Plan (BLM 2004) the BLM authorized 164,128 AUMs of livestock grazing on nearly 1.2 million hectares of public land. Greenhouse gas emissions from that grazing totals between 64,000 and 144,000 t CO_2_e/ year; far above the 25,000 t CO_2_e/year EPA reporting limit. Similarly, on the Wallowa-Whitman NF, GHG emissions were 98,000 t CO_2_e/year (GWP-20; Table 2). The GHG emissions from cattle grazing public lands are essentially unreported contributions to the climate crisis.

**Table 2:** The authorized extent of livestock grazing expressed as animal unit months (AUMs), greenhouse gas emissions (GHG), social cost of carbon (SCC), and income from grazing fees to the US Government for selected National Forests (NF) and Bureau of Land Management (BLM) Districts in Oregon

National Forest/BLM Resource Area	Number of AUMs	GHG emissions - GWP 20 (metric tonnes)	GHG emissions - GWP 100 (metric tonnes)	SCC - GWP 20 ($USD)	SCC - GWP 100 ($USD)	Grazing fees to the US Gov ($USD)	Citation, AUMs data
Malheur NF	132,000	115,500	51,612	$21,373,440	$9,543,600	$178,200	USDA-FS (2018)
Umatilla NF	49,000	42,875	19,159	$,934,080	$3,542,700	$66,150	USDA-FS (2018)
Wallowa-Whitman NF	112,000	98,000	43,792	$18,135,040	$ 8,097,600	$151,200	USDA-FS (2018)
Vale District BLM, Malheur Resource Area	423,672	370,713	165,656	$68,600,970	$30,631,486	$571,957	USDI-BLM (2019b)
Vale District BLM, Baker Resource Area	55437	48,507	21,676	$8,976,359	$4,008,095	$74,840	USDI-BLM (2019a)
Burns District BLM, Three Rivers Resource Area	150,472	131,663	58,835	$24,364,426	$10,879,126	$203,137	USDI-BLM (1992)
Burns District BLM, Andrews Resource Area and Steens Mountain Cooperative Management and Protection Area	98,045	86,104	38,476	$15,933,738	$7,114,682	$132,847	USDI-BLM (2004)
Lakeview District BLM	164,128	143,612	64,174	$26,575,606	$11,866,454	$221,573	USDI-BLM (2003)

Using the mean economic impacts of livestock removal from three western states provided by Taylor et al. (2022a, 2022b, 2022c) to extrapolate the costs from livestock removal from all public lands results in an estimate of $460 million in direct impacts and $1.4 billion in direct and indirect impacts. Social carbon costs of this land use exceeds the direct losses by $1.8 billion/year and the indirect and direct losses by $926 million/year. The proposed scientifically-based SCC values of Rennert et al. (2022) substantially increase the estimated benefits of greenhouse gas mitigation in comparison to estimates currently used in policy evaluation, and thereby increase the expected net benefits of more stringent climate policies.

Domestic livestock grazing on federally managed public lands across the western USA is damaging to the environment, more costly economically than beneficial, and is exacerbating the effects of climate change (Kauffman et al., 2022a). Yet their contribution to the food supply of livestock produced on federally managed public lands is relatively small; cattle grazed on these public lands account for <1.6% of all US beef production. While there remains great potential to conduct further and more refined analyses of the SCC of livestock grazing and similar land use activities on public lands, the exact estimate of SCC is less important than the fact that we need to achieve rapid, deep and, in most cases, immediate reductions in GHG emissions in all sectors of the US economy during this decade, including those associated with public lands grazing. Our analyses indicate that cessation of livestock grazing on US public lands is an environmentally and economically sound mitigation and adaptation approach to addressing the climate crisis. Doing so would also help to restore, and amplify the myriad of ecosystem services provided by intact wildland ecosystems. Indeed, applying the analyses and results presented herein to livestock grazing that is occurring at a global scale would surely illustrate the magnitude of this single land use and the importance of reducing ruminant grazing impacts in addressing the global climate and biodiversity crises.

## Supplementary Information

Supplementary Information

## Data Availability

Data on the aboveground biomass and carbon stocks of dominant semiarid ecosystems can be found in Kauffman et al. 2022a (supplementary information). Data on the number of livestock may be found in online databases provided by the USDA Forest Service (2018), the USDI Bureau of Land Management (2021) and in the supplementary information sections of both Kauffman et al. (2022a) and this paper. Data on emissions from livestock in the USA may be found in US Environmental Protection Agency (2018).
