# Supplementary material for: Forum: Climate, Ecological, and Social Costs of Livestock Grazing on Western Public Lands
Source: Environ Manage. 2023 Jul 14;72(4):699–704. doi: 10.1007/s00267-023-01853-6 (PMC10460345; doi:10.1007/s00267-023-01853-6)
Supplement: Supplementary file 1 — Supplementary Information [file 267_2023_1853_MOESM1_ESM.docx]

| Supplementary Information Table S1. The social carbon cost (SCC in $USD) per animal unit month (AUM) arising from the enteric fermentation and manure deposition of cattle on public rangelands. Results are based upon conversion of methane (CH_4)_ and Nitrous Oxide (N_2_O) to carbon dioxide equivalents (CO_2_e) using 20 and 100-year global warming potentials. We also report the SCC/AUM based upon published social costs of CH_4_ and N_2_O. Rennert et al. (2022) determined that the social cost of carbon is $185/metric ton for CO_2_e at a near-term risk-free discount rate of 2%. The USEPA (2022) also determined costs based upon a 2% discount rate, resulting in $1,600/metric ton for CH_4_, $54,000/metric ton for N_2_O, and $160 per metric ton for CO_2_e. The Interagency Working Group on Social Cost of Greenhouse Gases (2021) determined costs based upon a 3% discount rate, resulting in $1,500/metric ton for CH_4_, $18,000/metric ton for N_2_O, and $51 per metric ton for CO_2_e.   Total social cost of grazing from public lands is the sum from the average number of AUMs (cattle) grazing western public lands each year. | | | | | | | | | | |  |
| --- | --- | --- | --- | --- | --- | --- | --- | --- | --- | --- | --- |
|  | Rennert et al. (2022) | | USEPA (2022) | | | | Interagency Working Group on Social Cost of Greenhouse Gases, United States Government (2021) | | |  |  |
|  | GWP 20 | GWP 100 | GWP 20 | GWP 100 | N_2_O and CH_4_ | GWP 20 | | GWP 100 | N_2_O and CH_4_ |  |  |
| Methane emission- fermentation | $147.17 | $58.18 | $151.145 | $59.76 | $14.80 | $40.57 | | $16.04 | $13.88 |  |  |
| Methane emission – manure deposition | $3.18 | $1.26 | $3.27 | $1.29 | $0.64 | $0.88 | | $0.35 | $0.60 |  |  |
| Subtotal social cost CH_4_ emission/AUM | $150.35 | $59.44 | $154.41 | $61.03 | $15.44 | $41.45 | | $16.39 | $14.48 |  |  |
| N_2_O emission - manure | $11.57 | $12.86 | $11.88 | $13.21 | $12.60 | $3.19 | | $3.55 | $4.20 |  |  |
| Total social cost/AUM | $161.92 | $72.30 | $166.29 | $74.25 | $28.04 | $44.64 | | $19.93 | $18.68 |  |  |
| Total social cost – all public lands | $2.29 billion | $1.02 billion | $2.35 billion | $1.05 billion | $396 million | $630.0 million | | $281 million | $264 million |  |  |

| Supplementary Information, Table S2. The social carbon costs ($USD) of emissions arising from enteric fermentation and manure deposition on Bureau of Land Management (BLM) and US Forest Service (USFS) managed lands in the American West. The social carbon cost of $186/t CO_2_e is from Rennert et al, 2022; Table 1). The CO_2_e is based upon 20-year global warming potentials. At this level the SCC/AUM is calculated to be $161.92 (Table 1). Data are based upon the most recent livestock numbers available for these public lands. | | | |
| --- | --- | --- | --- |
| Year | BLM | USFS | BLM and USFS |
| 2007 |  | $871,541,848 |  |
| 2008 |  | $976,267,332 |  |
| 2009 | $1,272,910,925 | $988,018,353 | $2,260,929,278 |
| 2010 | $1,301,908,207 | $1,044,385,943 | $2,346,294,150 |
| 2011 | $1,335,051,773 | $1,007,052,696 | $2,342,104,470 |
| 2012 | $1,287,864,237 | $1,024,253,134 | $2,312,117,372 |
| 2013 | $1,219,344,227 | $944,578,455 | $2,163,922,682 |
| 2014 | $1,236,678,897 | $972,047,535 | $2,208,726,432 |
| 2015 | $1,299,764,224 | $1,022,914,541 | $2,322,678,765 |
| 2016 | $1,304,407,118 | $1,024,411,978 | $2,328,819,096 |
| 2017 | $1,314,619,574 |  |  |
| 2018 | $1,352,178,861 |  |  |
|  |  |  |  |
| Mean | $1,292,472,804 | $ 987,547,182 | $2,285,699,031 |
| SD | $40,848,685 | $50,815,073 | $67,766,313 |
| SE | $12,917,488 | $16,069,137 | $23,959,010 |

| Supplementary Information, Table S3. The social costs ($USD) of carbon related to the emissions arising from enteric fermentation and manure deposition on Bureau of Land Management (BLM) and US Forest Service (USFS) managed lands in the American West. Data are based upon the most recent livestock numbers available for these public lands. The social cost is based upon costs from combined CH_4_ and N_2_O emissions per AUM ($18.68/AUM; Table 1). The Interagency Working Group on Social Cost of Greenhouse Gases (2021) determined costs based upon a 3% discount rate, resulting in $1,500/metric ton for CH_4_, $18,000/metric ton for N_2_O, and $51 per metric ton for CO_2_e. The data from the Supplementary Information Table S4 in Kauffman et al. (2022) are in errata in that they were erroneously based upon a SCC of $35/AUM which was determined from for CH_4_ SCC for the year 2050 ($3,100/metric ton). | | | |
| --- | --- | --- | --- |
| Year | BLM | USFS | BLM and USFS |
| 2007 |  | $100,545,959 |  |
| 2008 |  | $112,627,679 |  |
| 2009 | $146,850,149 | $113,983,343 | $260,833,491 |
| 2010 | $150,195,438 | $120,486,224 | $270,681,662 |
| 2011 | $154,019,066 | $116,179,251 | $270,198,317 |
| 2012 | $148,575,247 | $118,163,590 | $266,738,837 |
| 2013 | $140,670,394 | $108,971,872 | $249,642,266 |
| 2014 | $142,670,219 | $112,140,859 | $254,811,078 |
| 2015 | $149,948,096 | $118,009,163 | $267,957,259 |
| 2016 | $150,483,726 | $118,181,915 | $268,665,642 |
| 2017 | $151,661,893 |  |  |
| 2018 | $155,994,943 |  |  |
|  |  |  |  |
| Mean | $149,106,917 | $113,928,986 | $263,691,069 |
| SD | $4,712,534 | $5,862,312 | $7,817,902 |
| SE | $1,490,234 | $1,853,826 | $2,764,046 |

**Literature Cited.**

Interagency Working Group on Social Cost of Greenhouse Gases (2021) Technical support document: social cost of carbon, methane, and nitrous oxide interim estimates under Executive Order 13990, <https://www.whitehouse.gov/wp-content/uploads/2021/02/TechnicalSupportDocument_SocialCostofCarbonMethaneNitrousOxide.pdf>

Kauffman JB, Beschta RL, Lacy PM, Liverman M (2022) Livestock on public lands of the western USA accentuate effects of climate change: Implications for mitigation and adaption. *Environmental Management* [https://doi.org/10.1007/s00267-022-01633-8](https://nam04.safelinks.protection.outlook.com/?url=https%3A%2F%2Fdoi.org%2F10.1007%2Fs00267-022-01633-8&data=05%7C01%7CBoone.Kauffman%40oregonstate.edu%7C52a47dcf8b00436081ee08db1f4c0ca3%7Cce6d05e13c5e4d6287a84c4a2713c113%7C0%7C0%7C638138183747749595%7CUnknown%7CTWFpbGZsb3d8eyJWIjoiMC4wLjAwMDAiLCJQIjoiV2luMzIiLCJBTiI6Ik1haWwiLCJXVCI6Mn0%3D%7C3000%7C%7C%7C&sdata=iZIWBBqb7GS7S0%2B4RZZ7o%2FSk0E3hWd%2B1MM300Mq716Q%3D&reserved=0).

Rennert K, Errickson F, Prest BC *et al.* (2022) Comprehensive evidence implies a higher social cost of CO_2_. Nature 610: 687–692 [https://doi.org/10.1038/s41586-022-05224-9](https://nam04.safelinks.protection.outlook.com/?url=https%3A%2F%2Fdoi.org%2F10.1038%2Fs41586-022-05224-9&data=05%7C01%7CBoone.Kauffman%40oregonstate.edu%7C52a47dcf8b00436081ee08db1f4c0ca3%7Cce6d05e13c5e4d6287a84c4a2713c113%7C0%7C0%7C638138183747749595%7CUnknown%7CTWFpbGZsb3d8eyJWIjoiMC4wLjAwMDAiLCJQIjoiV2luMzIiLCJBTiI6Ik1haWwiLCJXVCI6Mn0%3D%7C3000%7C%7C%7C&sdata=nSFywXLjh6szF%2BqK1h1Du%2B1joKGnBockQG%2BXYuh2VS0%3D&reserved=0).

USDA Forest Service (2018). Wallowa-Whitman National Forest Management Plan. https://www.fs.usda.gov/Internet/FSE_DOCUMENTS/fseprd584609.pdf

| Table 2. The authorized extent of livestock grazing expressed as animal unit months (AUMs), greenhouse gas emissions (GHG), social cost of carbon (SCC), and income from grazing fees to the US Government for selected National Forests (NF) and Bureau of Land Management (BLM) Districts in Oregon. | | | | | |  |
| --- | --- | --- | --- | --- | --- | --- |
| National Forest/BLM Resource Area | Number of AUMs | GHG emissions metric tonnes (GWP-20) | GHG emissions metric tonnes (GWP-100) | SCC (20yr GWP) | SCC (100-yr GWP) | Grazing fees to the US Gov |
| Malheur NF | 132,000 | 115,500 | 51,612 | $21,373,440 | $9,543,600 | $178,200 |
| Umatilla NF | 49,000 | 42,875 | 19,159 | $,934,080 | $3,542,700 | $66,150 |
| Wallowa-Whitman NF | 112,000 | 98,000 | 43,792 | $18,135,040 | $ 8,097,600 | $151,200 |
| Vale District BLM, Malheur Resource Area | 423,672 | 370,713 | 165,656 | $68,600,970 | $30,631,486 | $571,957 |
| Vale District BLM, Baker Resource Area | 55437 | 48,507 | 21,676 | $8,976,359 | $4,008,095 | $74,840 |
| Burns District BLM, Andrews Resource Area and Steens Mountain Cooperative Management and Protection Area | 150,472 | 131,663 | 58,835 | $24,364,426 | $10,879,126 | $203,137 |
| Burns District BLM, Andrews Resource Area and Steens Mountain Cooperative Management and Protection Area | 98405 | 86,104 | 38,476 | $15,933,738 | $7,114,682 | $132,847 |
| Lakeview District BLM | 164,128 | 143,612 | 64,174 | $26,575,606 | $11,866,454 | $221,573 |
|  | | | | | |  |
